# Understanding Coronavirus

**DOI:** 10.3201/eid2704.210152

**Published:** 2021-04

**Authors:** Xin Yin, Nicole M. Hackman

**Affiliations:** Pennsylvania State University College of Medicine, Hershey, Pennsylvania, USA (X. Yin, N.M. Hackman)

**Keywords:** coronavirus disease, COVID-19, infection prevention, pandemics, SARS-CoV-2, viruses

Accounts of pandemic illness are found throughout history (*1*). Despite advances in scientific knowledge and medical resources, society found itself repeating history with the global coronavirus pandemic. Though we have learned much about severe acute respiratory syndrome coronavirus 2 (SARS-CoV-2), many facts remain unknown and questions unanswered. In an age when information is easily shared globally, a demand for intelligent, understandable, and unbiased information exists, without which society experiences (and social media spreads) confusion, anxiety, and possibly panic.

Raul Rabadan, a professor at Columbia University and an expert and leading researcher in the field of genomics and systems biology, sought to provide much-needed accurate information. Understanding Coronavirus is a concise look at the recent history and epidemiologic, immunologic, and scientific concepts related to the pandemic (Figure). Organized as a series of questions and answers, his new book discusses many hot topics. Rabadan uses questions such as “How do we track back the origin of SARS-CoV-2?” to review the biology and epidemiology of coronavirus, the evolution and transmission of SARS-CoV-2, and key concepts involved in the ongoing pandemic and to offer comparison to previous pandemics, as well as those treatment and prevention options known at the time of publication.

**Figure:**
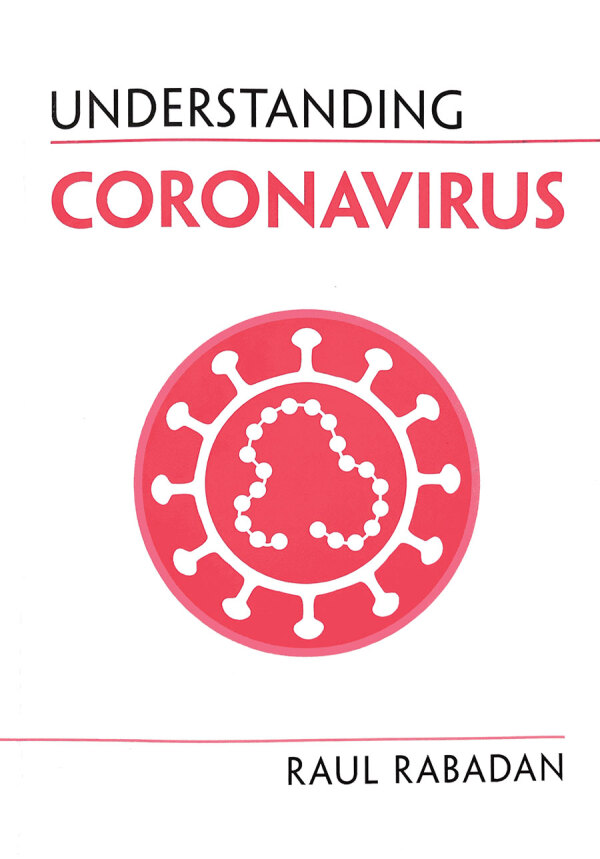
Understanding Coronavirus

Helping the reader to better understand viruses, Rabadan describes the basics of their biologic structures, origin, evolution, and spread in the first 4 chapters. He seeks to answer questions such as “What is a coronavirus?” and “How does the coronavirus enter cells and replicate?” Although he simplifies concepts as much as possible through illustrations and figures, it may still be a difficult read for those with little background knowledge. 

Chapter 5 details the coronavirus disease (COVID-19) outbreak by explaining the symptoms, at-risk populations, infection-fatality rate, case-fatality rates, and mortality rates. Other chapters are devoted to comparing and contrasting COVID-19 with the 2002–2003 severe acute respiratory syndrome outbreak and pandemic influenza, in particular the 1918 Spanish influenza. This historical information provides a sobering reminder that what is occurring is not unique to our generation. At the time the disease was beginning to spread in the United States, comparisons of coronavirus disease to seasonal influenza focused on similarities as contagious respiratory illnesses; further information revealed many differences between COVID-19 and influenza (*2*). 

The text concludes with a summary of common misunderstandings and suggestions for further reading for those interested in learning more about specific topics. We particularly liked the summary of common misunderstandings, which offers clear, straightforward answers to prevalent misinformation. However, the section “Updates at Press” could be improved by providing more context and citing specific sources for each finding.

Overall, Understanding Coronavirus is a well-written, well-organized, and informative book that would appeal to a broad range of readers, including epidemiologists, public health workers, university and medical students, physicians, and others interested in public health. Although the book lacks current details about treatment and vaccines, the scientific foundations introduced will be of great benefit for conceptualizing the pandemic now and in the future. 
